# Osteo-porosis*Read before the Fourth Semi-Annual Meeting of the New York State Veterinary Medical Society.

**Published:** 1893-12

**Authors:** Claude D. Morris


### OSTEO-POROSIS.*

BY DR. CLAUDE D. MORRIS.

In the presentation of this subject, I bring before you in a manner not at all satisfactory to myself, and I fear as will be viewed by you at the conclusion, a lame discussion on a disease not very prevalent in this country to any extent in an aggravated form; and yet which I believe is prevalent to some extent in certain localities in a mild form, and when existing in degree sufficient to warrant diagnosis. The baneful effects of the disease have made such inroads upon the animal economy as to baffle the skill and energy of the veterinarian to the extent, that Osteo-porosis is catalogued as one of the incurable diseases affecting the lower animals. However, I cannot believe it is a disease whose only sequel should be ultimate dissolution.

The literature bearing on this disease is not abundant. In 1860 Professor Varnell wrote an interesting article on Osteo-porosis, and it is claimed that Varnell was the first to describe the disease. Later, his article appeared in the *Veterinarian* of that year. About 1870 Sir James Paget relates before the Pathological Society, London, his experience with a disease termed by him as Ostitis deformans, and from the description he gives, stating symptoms, post mortem appearances, &c., I think there is no doubt as to the identity of the disease previously described by Varnell. Again, in Dr. Lippincott's report of 1875-6, Phil., relates the history of a disease under the head of "Ostitis deformans," which bears a striking resemblance to that of Osteo-porosis. Also Lane, in Guy's Hospital Report, Treasurer of the Pathological Society, London, 1886, describes at length Osteo-porosis, and I will here quote one paragraph from his very interesting and valuable paper in that report, before discussing the disease, from American pathology. Lane holds that the thickening of the skull is a process of repair, and not a direct result of the disease, and says that in Mollitis ossium the thickening of the skull is dependent upon an endosteal, and later upon a periosteal, deposit of decalcified lamellæ of fibrous tissue, this being not a direct product of the condition, but an effort on the part of the organism to oppose and limit the baneful influences of the process of decalcification in so far as it affects the cranial vault. He applied the same argument to the analogous condition of the skull present in Ostitis deformans, rickets, and congenital syphilis.

* Read before the Fourth Semi-Annual Meeting of the New York State Veterinary Medical Society.

What is Osteo-porosis, as understood on the American Continent? It is a non-inflammatory disease of bone, characterized by defective action in one or more of the joints, affecting one or two of the limbs in an alternate manner, a much tucked up condition of the abdomen, an arched spinal column, a straightening of the limbs, and as the disease progresses an enlargement of the facial and sub-maxillary bones, with defective mastication, accompanied with tenderness upon manipulating the bones of the face. During the entire stage of the disease the pulse remains about normal, with slight variation of temperature, obscure pains, metastatic in character. resembling that of rheumatism.

I beg to give you a brief history of this disease. as was manifest in these specimens before us. On the 14th of last February I was called to examine a two-year-old stallion suffering with lameness in the right fore extremity. Examination revealed tenderness and some degree of heat in the heel. As the animal was being conditioned for fast work, I thought it not at all strange under the circumstances that he should bruise the heel, which seemed a valid cause for his lameness. Therefore I diagnosed the trouble, Navicular Arthritis. On the 10th of March I was called again. The lameness had then left the fore extremity. and was at that time very pronounced in the left hip. It was at this time that I began to mistrust the impending trouble, whereupon a thorough examination was made which proved beyond doubt my suspicion.

This case was watched daily from this time till May 25th, when it was deemed as only humane to end the sufferings of the animal. During this period the disease presented the following symptoms: A constant degree of lameness in some one or more of the extremities at the same time; the peculiarity of which was, when lame in two limbs, at the same time, it would be that of the hind and fore limbs of the opposite sides. The greatest degree of lameness existing was in the left hind leg, which became straight, as the disease progressed, to such extent that a straight line drawn from the trochanter major externus to the os pedis would touch the entire length of all the long bones of the leg. A general atrophy of the muscles of the limbs, and at times an inability to rise without assistance. Appetite good, but towards the last there was a disinclination to masticate ordinary foods, gruels being administered instead; the bowels remaining normal throughout; urine natural in quantity and color. Post mortem examination presented a most interesting revelation. I will relate a few of the more prominent lesions. The animal was bled for the purpose of dissection. The muscles of the legs were paler than natural, and maculate. In disarticulating the limbs from the trunk, and especially the bones of each limb one from the other, the interior of the joints presented a contrast. The articular cartilage was of a purplish hue, much thinner than natural, and in some instances entirely lost, leaving the margin of the articulation at that point quite exposed.

In others the articular cartilage was of natural thickness, but easily detached from the bone. In these the synovial fluid was darker in color, and in some instances streaked with blood. The synovial membrane was much thickened at the parts most vascular, then again these same structures in other joints were much paler than natural. The periosteal covering of the long bones was quite vascular and easily detached, especially the bones of the skull. Some of the flat and irregular bones were soft, and could be readily cut with the knife; one very noticeable feature being that all ligamentous and tendinous attachments were easily separated, bringing portions of bone as they were torn loose.

These attachments were much discolored, indicating that local inflammation, or extravasation of blood, had taken place. I am of opinion that there must have existed considerable inflammation in the soft structures affected, and was sufficient cause for the manifestations of lameness which existed in certain articulations.

Another post mortem characteristic is that the weight of the bones is greatly diminished, apparently one-third of their weight being lost. The increased size of the medullary canal is well marked in the long bones; and what seems to be a complete breaking down of the cancellated structure is visible throughout. The same degree of destruction exists in the compact tissue. The haversian canals, the lacunæ and canaliculi appear as homogeneous.

Another noticeable feature of this disease presented itself while cleaning the bones; perhaps due to their extreme softness. The different processes (epiphyses) were detached from the bone. The application of the slightest force upon a tendon or ligament was sufficient to effect their removal. Contrasting a healthy bone with one affected with this disease, we observe that at the ends of the long and flat bones the disease has permeated the compact as well the cancellated structure. The increased size of the haversian canals is very patent, as are the foramina for the passage of blood vessels; the same existing in the periosteal membrane, causing the bones at the extremities to assume a honeycombed appearance. Thus I might proceed at length to narrate post mortem lesions, believing, however, that a few briefly stated facts will prove more interesting than a paper treating at length on all the details.

From all sources obtainable in the literature treating on Osteo-porosis, and from limited experience, the causes and morbid development of the disease is still enshrouded in more or less mystery. However, I am forced to believe from crude reasoning that osteo-porosis consists in a degeneration of the normal development of bone; the process being assisted primarily by decalcification, a gradual absorption of the calcareous materials, a diminution in the function to assimilate bone producing foods, and latterly, an unnatural development of the vascular and fibrous tissues of bone, devoid of the true elements of osseous and cartilaginous structures. I base this reasoning on one fact, that osteo-porosis is a disease of development, which only occurs during the growth of the animal, there being (so far as I can learn) no record of the disease in the adult animal.

If osteo-porosis is a disease whose incipiency originates in faulty digestion, which must necessarily entail a deranged absorpant system, thus attenuating the functional activity of assimilation, would it be unreasonable to suppose that the osseous structures ought to suffer quite as much as any other structure or vital organ of the economy? If food and oxygen are the source of all physical energies when properly appropriated in their relation to the maintenance of animal life in obedience to Nature's divine laws, there is then but one expectancy. It seems to me that therein lies the panacea for all physical ills.

As I view the azure and crimson twilight in this the evening of the nineteenth century in the great work wrought in medical science, preparatory to taking her place in the front rank at the dawn of the twentieth century, as the Science of Sciences, in its benefactions to animal existence high or low, I cannot forbear the belief that within the next few decades disease must succumb in the conquest now waging as between the mysteries of insidious disease, and the knowledge to which the medical science shall then have attained. When the declaration, so oft repeated by physicians, which is but a warning to the "animal" and an acknowledgment of human weakness. There now remains no further aid that man can afford. This sentence shall then give place to a broader, more intelligent, and of complete mastery over disease, "Go live in peace your allotted time."

*Dr. Morris* added.—With this paper I have a few specimens which I thought might prove interesting. In the first place, you will notice in my paper I stated it was early in March when I saw the case first, and the horse had not been lame for about ten days. Perhaps the first or second day of March was the first that the animal was noticed going lame. This colt could go out and trot in better than thirty; he was a little past two years old, and he was bred in north-east Pennsylvania, sold to some stock people down in Lexington, Ky., brought back to New York last January, and sold there at a very fair price. He was taken to a stock farm in the southern part of Dutchess County, and on the 15th day of May we destroyed the animal, and in that short time the disease, which is so apparent here in this skull, was fully developed. I have since seen several specimens of the disease, but I consider that one as good as I have seen. In going over the bones, and especially at the femur, a very noticeable feature is its extreme light weight, which is so marked that by simply taking the bone in your hand, without seeing it, you would not believe that it was a bone; and the epiphyses of the bones, the different developments for the attachments of muscles and ligaments, you can see that as the muscles and ligaments were torn loose the portions of the bone have gone with it, breaking off, especially where the glutæus muscles were attached, on this projection, easily gave way as we tried to remove them; and also after the bone had been cleaned and laid out in the sun to dry, the extremities dropped off very easily; and other portions of the skeleton, some parts which have not been out in the sun at all, are seemingly going the same way; all the portions are coming off very readily indeed. It seems to be one of the characteristics of the disease that the bone absolutely disintegrates, and that all the parts which have been homogeneous and in perfect contact in the healthy bone, give way very readily. Then when we examine the interior of the bone we notice again the ravages of the disease. The cancellated structure has become entirely broken down, and in examining the compact tissues with the naked eye you can very readily see the disintegration, particularly of the Haversian canals, and there is but very little solid, compact tissue about the bone; and this was in an animal, you must remember, that was suffering only 90 days with the disease; and it seems to me that everything must have been very opportune for the disease to permit of its producing the ravages here manifested in an animal which 90 days prior was apparently in perfect health. We find again that the disease permeated not only all parts of the bony structure, but even the teeth were not exempt, as the crusta petrosa show manifestations of the disease as much as any other bone, or any other part of any bone, and it is especially noticeable in the alveolar processes, that it comes right out easily, spongy in nature, very light; the specific gravity is comparatively nothing, whereas in its normal state it has a considerable specific gravity, and is quite intact and homogeneous and solid, while in this instance it is easily detached and very light in weight.

*The Chair, Dr. Wende.*—Dr. Morris' very interesting paper on this very important subject is now open for discussion. If any of the members have similar cases we should like to hear from them.

I have seen quite a number of these cases. In 1889 I met with five in the city here. Four out of the five were horses that were brought here from the West. One was a horse that was raised right out at the north end of the town here, and where we have the finest limestone quarries in this part of the State. He was raised by a party that owns the quarries, and raised right on that limestone. He was ailing to my knowledge for about a year. My attention would be called to him and I would find him lame in one leg or another. He would be laid up and rested for about a week, when he would do about a week's work again, and then have to be laid up again. Finally, I think in May, 1889, I had a call out there, and one of the men told me to look at the colt again. He was five years old then. I had not seen him probably in six weeks or two months. At this time I recognized the disease by the enlargement in his face. I ran a horse-shoe nail right into the side of his face. The skin was the hardest part to puncture. I told the man what I thought the disease was, and that I thought him incurable. About a month afterwards I was called out again. I found the horse extremely lame. Another veterinary surgeon had been called in, and he advised the bathing of the limbs for a week, two hours each day, at the end of which time he would come out and put on a blister. Well, I told them to let the man proceed; that I didn't believe it would do any good. About a week or ten days afterwards I was called out there again. There lay the colt. They couldn't make him get up. They asked me to come out and sling him. They had always been good customers of mine, so I went out to sling him up. When I got there sure enough the other Vet. had blistered the horse. They told me that about two hours after the blister had been applied the horse lay down and never got up afterward. I used to be out there two or three times a day, or an assistant, and we raised the horse up. He would not touch a foot to the ground. I think we did that four or five days, until he commenced to get bed sore, when they allowed me to destroy him. I have the skull of that horse at my infirmary.

Then, I think in January, 1891, I had a horse in my place, a boarder, the only boarder I had, and one that I boarded almost three years. This horse was five years old, and Kentucky bred. He was taken sick with influenza. I treated him, and got him over it all right apparently, but seemed to have a sequel that I diagnosed as rheumatism. He would be lame in one leg and then the other. All the treatment that I could give him wouldn't do him any good. If I got him straightened up so that he could walk fairly well, and the man take him out and drive him about an hour, which probably would happen once a week, then he would be worse again. I kept him there until the latter part of March. I went off to Toronto to the examinations, and was gone about a week. When I returned and saw the horse, I recognized at once that his face was enlarging, and tested his face with a horse shoe nail. You could run it into his face without any pain at all, and strike the nail with your finger so that it would "ding," as the stableman said. Well, having treated that horse then for nearly three months for rheumatism, I didn't dare go back on my first diagnosis. Before I went away the owner had been ready to sell him, so now I advised that he sell him if he got a chance, which he did. He sold him to a homeopathic physician in town here, who said he could cure him. I felt satisfied that he could not. At any rate he took the horse, and took him to another stable to board, in which the horses are kept on the second floor. He gave him his pellets, and sure enough the horse commenced to improve, and is being driven on the streets here to-day. The doctor attributes the cure, of course, to the use of his homeopathic pellets, while I attribute it to the surroundings. He never knew but that the horse was suffering from rheumatism. I never told him that I had changed my diagnosis in the case.

*Dr. Sutterby.*—Had his face returned to its normal condition?

*Dr. Wende.*—His face is somewhat enlarged yet, but I do not think it is so much enlarged as it was at the time he left my stable.

*Dr. Sutterby.*—You haven't had an opportunity to put a nail into it to see whether it would "ding"?

*Dr. Wende.*—I have not—well, I have had the opportunity, but I wouldn't try it for fear I might give myself away. This horse, also, gradually seemed to lose appetite or power of mastication, and he was very thin when he left my stable. He certainly eats all right now, and is in good condition.

*Dr. Morris.*—You say that these horses that you spoke of first, out here, were on a limestone soil?

*Dr. Wende.*—One horse, the five-year-old horse, was raised in a barn that stands right on the limestone, and has all limestone surroundings.

*Dr. Morris.*—The drinking water undoubtedly ran through lime water?

*Dr. Wende.*—Yes; it was all natural water then; they had no city water at that time; they have it now.

*Dr. Morris.*—I know that one of the causes of the disease—when you see it in that form, it shows a want of lime; the calcareous materials are wanting entirely; it is one of the elements which go to make up a bony constituent, and that is just what it is deficient in.

*Dr. Sutterby.*—The hard part of bone?

*Dr. Morris.*—Yes, the lime, or earthy salts, which go to make up the bone; and that would be one of the recommendations that I would suggest, that the horse be put on a diet—or put in a section where he would be benefited by receiving more or less of just those very elements, what would seem to be deficient.

*Dr. Wende.*—This was right across the road from the largest quarries we have in the western part of this State, and the quarries there are pure limestone, thirty feet deep at least. I would also say that all these horses that I have noticed with the disease have been kept in stables with generally a plank floor, but the floor resting down on the ground with no ventilation under it.

*Dr. Sutterby.*—And effete matter had accumulated for years?

*Dr. Wende.*—With the exception of my stable. My stable is well ventilated, and there is ventilation under the stable.

*Dr. Sutterby.*—Well drained?

*Dr. Wende.*—Well drained, every way. This horse was brought here from Kentucky, I think five or six months before he was taken down with the influenza, and probably the other disease existed in his system to a certain extent before it developed at my place; and then, too, during the winter he was at my place the snow was very deep, and there certainly was no ventilation through under the barn then. I would like to ask Dr. Hoskins whether they have had any like experience in Philadelphia.

*Dr. Hoskins, of Philadelphia.*—I might say, Mr. President, that the disease is quite prevalent in our neighborhood. It is not the exception by any means. We rather feel that it is one of the current diseases that we are dealing with there right along. I do not know that our location has anything specially to do with it. It is also quite well known that among Shetland ponies it is extremely common, and they are never sheltered at all—often assuming an enzootic form among them, and in certain seasons, as if the forage at that particular season lacked the essential ingredients for building up the bony structures of the body. I have known the same thing to occur on a farm, within about twenty miles of Philadelphia, among a large number of running colts, assuming an enzootic form in a particular season. One season I think there were about thirty cases of it, there being only about sixty in number of the colts altogether, and it was attributed to the fact that the season, either from excessive dryness or some other peculiar atmospheric condition, the forage of that season did not contain the necessary constituents for building up the bony structures. Among running stock it is quite common, and in the neighborhood of the running tracks, close to Philadelphia, there are many cases of it. I have seen no conditions under which it would not develop. I have, like Dr. Wende, thought that the board floors were conducive to its development. While Dr. Morris has called it a non-inflammatory disease, I have seen it ushered in by all the evidences of inflammation that one would want—swelling of the bridge of the nose, heat, tenderness, and all the essentials of an inflammatory action. I have seen the same thing have its origin in joints, presenting all those same symptoms of heat, swelling, tenderness, and evidence of an undue amount of blood circulating in the parts. And I think it is one of the diseases in which there is no form of lameness that it will not assume ; and it is, I think, the one disease above all others that I would be very frank in saying I have made more errors of diagnosis in than in any other direction. Looking upon it as a sprain, or an injury, I have, some months afterward, been called back to find the same lameness in probably another limb, only to recognize that it was of the character of osteo-porosis. We have found that the lime water treatment is a desirable treatment, and you have present with you here to-day one of your new members, who witnessed a remarkable recovery under the lime water treatment, and I hope he will take the floor and tell you about it. It was of interest to us all in eastern Pennsylvania, and I think would be equally interesting to you. I do not feel that we understand the disease very thoroughly, but the best treatment I have found for it is to turn them into grass ; take them off all grain and all hard food and turn them to grass Under that treatment I have had a number of recoveries, both in ponies and in mature horses ; but from anything in the line of therapeutics I have had, with the exception of lime water, no results that would warrant the continuance of the plan of treatment adopted. I have seen the application of blisters over the parts where it had started check it, and seem to hold up for a time the progress of the disease at that particular point. It has been noticed so far as the influence of lime countries is concerned, that the disease seems to prevail in lime districts to as great an extent almost as in other sections ; and in sections of country where limestone does not prevail they do not seem to have any more of it than they do in a limestone country ; but there does seem to be a very great factor in the atmospheric conditions that prepare the forage for horses ; and in certain seasons when we feed the oats and the food of the preceding one, we find a much larger number of cases of osteo-porosis than in years before, which would point very strongly to the likelihood of the food being a very large factor, when from any cause it lacks the essential materials for building up the osseous structures.

*Dr Morris.*—I would like to ask Dr. Hoskins what he bases his reasons on that osteo-porosis is an inflammatory disease? You have taken the temperature in those cases I would infer, and the temperature indicated that it must be of an inflammatory nature, or that the heat, locally, was what you drew your conclusions from.

*Dr. Hoskins.*—I draw it solely from the local lesions. As has been well said, by the writer of the paper to-day, the pulse is rarely ever disturbed, either in frequency or in number, but you will find all the local lesions of an inflammation in many instances—not in all. I think though that so many of them start along the spinal column—at least so many that I see—along about the first lumbar vertebra or the last dorsal vertebra, that the thickness of the muscles and the other tissues surrounding the part make it impossible to detect the heat, the tenderness and the soreness ; and one of the earliest symptoms that I have noticed in a great number of cases is, that when you pandiculate them they show a great tenderness, and show the tenderness when you pandiculate over where the saddle rests quite as quickly as back, where they usually yield to pressure. As a rule, when you get beyond the lumbar vertebrae, going to the anterior portion of the horse, they do not yield on pressure there, but they do yield very frequently in osteo-porosis, and I would like to state that one of our veterinarians in Pennsylvania claims that he can diagnose the disease if there are any lesions of the head whatever, though not discernible to the eye, by listening to that horse chewing grain, that he invariably detects a hollow sound when he is chewing his grain ; that he will make a diagnosis from that and risk the future to prove the correctness of it. I have been waiting for opportunities to test that myself, but have not as yet had those cases that would give me the chance to do it. Most of the cases I have seen have been either of long standing, that have come under my notice when they were beyond the necessity of resorting to any obscure method of diagnosis, the lesions presentable to the eye being so conclusive that it was unnecessary.

*Dr. Morris.*—How old were the oldest animals that came under your notice that were affected ?

*Dr. Hoskins.*—I rarely ever see one beyond the age of eight, and I have seen them as young as six months.

*Dr. Morris.*—As I stated in the paper, the literature bearing on the subject is so meagre that it was very difficult for me to find histories of instances, although in looking over the American Veterinary Journals I found one instance of a paper, by a gentleman in the State of Pennsylvania, stating that in one stable, I think, half a dozen or eight cases occurred, and he spoke of it as being a sort of an epidemic in that stable, and he gave a few reasons in support of that view, whereas on the other hand, speaking of the inflammatory character of it, I based my conclusions more upon history than upon experience, especially the Transactions of the Pathological Society of London, from papers that were submitted there on that disease, which was diagnosed more as ostitis deformans, but which undoubtedly is one and the same disease. They say there that it is a non-inflammatory disease, and the reason why they say so is that the clinical thermometer is the true test of an inflammatory disease. Now, if you find inflammation locally, and it does not raise the temperature constitutionally, then we can say we have an inflammatory disease. For instance, we may hurt a part, we may bruise a part, break a limb—we have local inflammation, but have we an inflammatory disease ? It is merely a matter of discretion, of how different men agree, as to what inflammatory disease is, whether one constitutionally speaking or one locally speaking. Inasmuch as the disease affects the whole system—every bone, I believe, is affected in a well-developed case of osteo-porosis—inasmuch as the whole system is affected and the clinical thermometer does not indicate a rise of temperature over one-half degree—that was my experience—and then I was led to agree with the former men on the subject, that it was practically a non-inflammatory disease. Still, that may be an error.

*Dr. Hoskins.*—It may, Mr. President, be a non-inflammatory disease, but certainly when you get those cases with the disease showing itself along the spinal column, and with that thickening on the inner surface of the canal, you will get a rise in temperature, 102, 103, 104. Now, whether that could be directly attributed to the changes or alterations in the bone, or whether it is rather due to the changes in the bone producing other changes, of course is open for question. Now, I don't quite agree with the Doctor wholly as to the statement that all the bones are affected, because I have witnessed some three cases where the lesions were confined entirely to the head, and in each one there was a complete recovery so far as usefulness is concerned ; and I know of one horse that was recently destroyed for another disease that had osteo-porosis of the head for a period of eight years and never missed a day's work from it—never missed a day's work. For five years he was under my own observation, and I could trace him back three years when the head was in the same shape. He was destroyed a few months ago for another cause not connected with osteo-porosis. I have his head, which I secured, and it is a fine specimen of osteo-porosis, the horse being about thirteen years of age. I know of the same in a Shetland pony, where I advised his being turned out for a period of six months. There was another case where there had been some three Shetland ponies on the same farm, all afflicted ; two of them had been destroyed by other veterinarians prior to my being called there for advice on the third one. I advised his being turned out, and was blessed with the good fortune to see him make a good recovery, and he has been driven by the children who own him for the last three years, the lesions entirely confined to the head. And there has been in that case a return of the head to almost its normal condition. An expert, or one who had seen the case before, would detect still a slight enlargement over the superior nasal bone, but I think the average judge would say, "Well, he is probably fuller at that point than most cases are." Now, certainly without any evidence of any breaking down, any lameness, or any loss of power in any other part of the body, the lesions being confined entirely to the head, we could hardly say that all the bones were affected. Recent writers have taken the position—and some of the strongest in veterinary literature—that rachitis and osteo-porosis are identical.

*Dr Wende.*—Speaking of all the bones being affected, I have one very fine specimen, in fact the finest I have of osteo-porosis, where there is presented on the inferior maxilla a well-defined line of demarcation, if I may so term it, showing where the disease existed and where it did not exist. It only existed on about two-thirds of the inferior maxilla, on both sides, running back about two-thirds of the way and then ceased. It was very well developed too where it did exist. It is the only specimen of the kind that I ever saw marked like that.

(To Dr. Morris).—Did you notice anything of that kind on your specimen?

*Dr. Morris.*—In this case of mine I examined all the bones of the skeleton—took particular pains to examine them all—and I have failed to find a bone that does not show the ravages of the disease. The scapula of the right fore-limb was as light as a fan, to hold it in your hand, and I supposed was in the box which I brought here, but find that the young man who put them up for me neglected to put in the scapula. I do not mean to say that no case could exist wherein all the bones were not affected I can readily see how a horse of stronger resistance could throw off the disease and limit its baneful influence ; but here was an instance where the structure all gave way, the animal being only a little over two years old. Possibly the older the animal becomes the greater would be his resistance to the disease and his power to limit its effect. I can easily see how that may be accomplished ; but in a young animal like this, where the disease was well developed and well under way, lame in all four extremities, the arched back, the curved cervicle, and sore from head to foot—I think everything then is pretty nearly affected. However, this patient was diseased from beginning to end ; I cannot exempt any part. I should like to hear from the gentleman that Dr. Hoskins speaks of about this lime-water treatment.

*Dr. Willganz.*—I am the man that Dr. Hoskins referred to. I was a student at the University of Pennsylvania at the time that this animal was sent there. The case was placed in my charge. It was sent from Gloucester, N. J.—from the race track, having been there for about six months, worked every day, and said by a doctor down there to have been at one time lame all over. Shortly afterward they called for our ambulance—probably two weeks afterward—and said that the horse had broken down. I went over with the wagon for the horse. I recognized a tenderness at the fetlock, a good deal of heat ; in fact, there was pain on manipulation. The animal could hardly walk into the ambulance. We took him back to Philadelphia and led him into a box stall. The next morning when we went through the hospital, about five o'clock, he was standing. At seven o'clock he was down. His temperature was taken, and there was an elevation. We got slings under the animal and raised him, leaving him that way for about a day. Then we concluded to remove the slings, and he stood for probably three days. Then he went down again. This was the first time I had ever heard of this lime water treatment; it seemed to have quite a run through the city of Philadelphia, where there were a great many cases of this disease. It was tried, and at first there was little or no change. The owner came over several times and said he was sick of paying the hospital bill on the horse and they better destroy him. He was told not to be too hasty, and at last he turned round and gave the horse to the University. Well, they continued the lime water treatment for a while, and the owner shortly afterward got a letter stating that his horse was all right. He then returned, paid the hospital bill on the horse, and they allowed him to take his horse. This horse had the usual swellings around the head and the inferior maxillary bone, and, as had been diagnosed before, he had been lame in the different extremities, and rest usually was a cure. Now, in regard to the disease being localized to the head alone, I can say I have seen cases that were destroyed at college where there were no other evidences of the disease than those about the head, while in other cases there were well marked signs of osteoporosis throughout the body.

*Dr. Hoskins.*—I can well understand in this case that Dr. Morris has reported here, why the entire body was afflicted. The animal was but little over two years of age; the process of calcification of the bones had not been completed. As he well denotes there, in the inferior extremity of the femur, where one of the centers of ossification or calcification resides, he has the whole of the inferior extremity, or that whole center, to drop off. Why ? Because that had not been a completed process; and he certainly had a very acute case of the disease, and it found an easy road to attack the entire body, because the completion of the bony structures was yet to take place. In addition to that, this process had been materially hindered by the fact that this animal was put to more or less severe work ; and these factors will largely account for the fact of his having so acute an attack of the disease and its running so short a course.

*The Chair.*—I would like to ask Dr. McLeod whether he ever met with any of these cases in this city as a student or a practitioner.

*Dr. McLeod,* of Buffalo —Not but the one, and I presume you remember that case, of a large colt out at the street car barns. I am sorry to say there never was any post-mortem held in that case; he was simply taken out and shot and carted away. But his hind extremities, as Dr. Morris says, were straight, perfectly straight. This horse was two years old, and he measured 16.3 forward and 17.2 behind. There was three inches difference between his withers and his hips, One side of his face was swollen to some extent only; the other side was natural. That is the only case I ever remember seeing.

*The Chair.*—Was he lame ?

*Dr. McLeod.*—Why he was all out of shape. You could hear the bones in his hind leg; you could hear a noise; used to thump every time he walked, as though they slipped out of joint.

*Dr. Sutterby.*—Luxation. What amount of lime water would you advise giving to a horse two years old?

*Dr. Hoskins.*—Give it *ad libitum.* A very good plan is to put a barrel in front of him, throw some lime in it, keep changing the water, and let him drink. What they do at Gloucester, when they get them down there, is to put them in front of a barrel of lime water and let them drink. If you don't stir the water—you know, of course, there is just so much that can be held in solution, and they may drink just as much as they want. There have never been any bad results follow from the adoption of that plan with them. Dr. Willganz says the colt at the University had a bushel of lime to a barrel of water.

*Dr. Wende.*—Dr. Drinkwater, do you ever meet any cases in Rochester? You have been there quite a long time.

*Dr. Drinkwater*, of Rochester.—I have had three or four cases, but I can't say that I have had any where the joints were affected, or any other part of the body than the head. I had a very decided case at Albion a few years ago, in a mare that was ailing for years—must have been 12 or 13 years of age; and she was perfectly sound in every other respect except her head, and that was in the same condition as this specimen that Dr. Morris shows, and the teeth eventually fell out. The animal was destroyed. I have the specimen, and I think the teeth are all out on one side, but not all on the other.

*The Chair.*—Couldn't that have been a case of osteosarcoma?

*Dr. Drinkwater.*—No, osteoporosis.

*The Chair.*—Not osteosarcoma as we were taught at college, what now is known as Ostitis deformans.

*Dr. Drinkwater.*—Oh, no, it wasn't that.

*The Chair.*—I have several specimens where the teeth have fallen out.

*Dr. Drinkwater.*—Yes, and the bone became solid.

*The Chair.*—I could reach in and take the tooth right out.

*Dr. Drinkwater.*—Not the same as you find in osteosarcoma.

*The Chair.*—I have seen a great many cases of osteoporosis, but I never saw a case where the teeth fell out, or where you could pull them out with the hand.

*Dr. Drinkwater.*—Well, I could in this case.

*The Chair.*—But in sarcoma I have.

*Dr. Hoskins.*—I might state, too, that the age of this horse was rather against the probability of its being osteoporosis, and would make it more likely to have been osteosarcoma. Of course the microscope would have been the only thing to determine. You all remember the remarkable case of "Prospero" that they had up in New York State a number of years ago. In that case you remember the controversy among the veterinarians who had examined him. He was a noted runner, a stallion I think. His trouble had been diagnosed by a veterinarian from New York City as osteoporosis, and subsequently another veterinarian dentist was called n and said it was nothing but his teeth, and he removed two of them and hid them, or took such good care of them no one was ever able to see them—and failed to find any lesion of the teeth, any caries of them, or of the alveolar cavity, and he pronounced the horse, if you remember, on the road to recovery. He continued to lose flesh, and when he died I think he weighed 550 pounds. His head was removed to New York City and examined, and it proved to be a case not of osteoporosis, but osteosarcoma. It took the microscope to determine the nature of the lesion. All the symptoms, as far as could be observed, pointed to osteoporosis. I believe I have never seen a case develop after eight years of age. I have seen horses 12 years of age have it, but they had had it for years ; it localized itself in the head.

*The Chair.*—Dr. Hinkley, we have not heard from you.

*Secretary Hinkley.*—Unfortunately for me, I was absent during the reading of Dr. Morris' paper. So far as my own experience is concerned, I have had very few cases, not over 25 or 30, in my practice. Some have made very nice recoveries; others have die[illegible] I notice that the cases where turned out as incurable and left to themselves made a beautiful recovery, not through any shrewdness on my part, but simply observing them, so that I have been in the habit recently of ordering these horses turned out. I had one case in particular of a Waveland Chief colt. I should say we elevated him in the hospital about three or four weeks ; elevated him three times a day to eat, and as some one remarked, he was lame all over ; the inferior maxilla was very much enlarged ; the joints were all enlarged ; the peculiar arch in the back ; and he was a sorry looking object. It came on in the month of April, and a little later the pastures became very good, and we took him in the ambulance out to pasture, turned him in there, and he was sold last fall at a sale in Cleveland, and brought four hundred and some odd dollars, and went West as a stock horse. That was only one of the cases that made a good recovery. I fully agree with what little I have heard Dr. Hoskins say ; I think that in the treatment rest is one of the essential points. The lime water treatment is entirely new to me ; I never heard of it before.

*Dr. Morris.*—I would like to say a word. Prof. Richardson of the Toronto Veterinary College is with us, and perhaps he could give us some light on this subject. I would ask the courtesy of the floor for Prof. Richardson, if he will kindly speak to us.

*Prof. Richardson.*—I won't attempt to speak from the medical point of view at all, but two or three points of interest occur to me in connection with the purely chemical side of the question. So far as I understand, you have before you a process of decalcification of the osseous tissues. The surroundings of the cases, I think, might throw a good deal of light on the causes of the disease. For instance, you have mentioned certain cases in which young animals have been put to excessive work at an early period of their growth. In others you have an unsanitary condition, improper ventilation. You have also the fact that this disease is not limited to certain localities, but you will meet it on the blue grass of Kentucky, you will meet it elsewhere, where there is no limestone, and you will meet it even, as your President has said, on so essentially a limestone formation as lies north of Buffalo here Now, it cannot be said, speaking chemically, to be due to a lack of lime in the food, because if this were so, those portions of the country which are situated upon limestone ridges, where there is plenty of lime in the soil, ought certainly to be free from the disease. From the glance that I had at some portions of the bones, it strikes me that it is due not so much to a lack of lime salts as to an inability to digest or assimilate them into the system. It seems to me that it is entirely due to decalcification, or, in other words, a taking up of the calciferous salts that have already been deposited. Now, we know that one of the greatest agents at our command in the animal economy for carrying the calciferous salts is carbonic acid. We see this in cases of rheumatism, where there is a lack of carbonic acid in the blood, and where the calciferous salts are deposited. If at any particular point in the body there should be, due to local innutrition or other causes, or excessive use of certain of the muscles, an undue preponderance of carbonic acid in that vicinity, and the system, owing to unsanitary conditions, or local conditions, is not able to eliminate the carbonic acid rapidly, it may be eliminated through the calciferous salts taken up by the excess of carbonic acid—may then be eliminated through the channel of the kidneys ; and I think if you were to make—I speak this now from theory—if you were to make a careful examination of the urine you might find that the phosphatic salts were very much in excess. From these two chemical points you might reason—infer—that the disease might be met with the lime treatment ; that is, throwing a large excess of an alkali into the system, and in that way neutralize the action of excessive quantities of carbonic acid. At the same time I would also point out the advantages of sanitary surroundings; of placing the animal out in the open air, where the rapid elimination of the carbonic acid, or probably other acidulous salts, which ought to be eliminated, can be eliminated more readily. These points seem to me to be intimately connected with the disease as presented this afternoon.

*Dr. Hoskins.*—I would like to ask Prof. Richardson how he would account for an enzootic outbreak among colts that were running at grass and not under cover or shelter at all.

*Prof. Richardson.*—That might be due to causes already pointed out; that is, the change in atmospheric conditions, with the changing food.

*Dr. Hoskins.*—I might state that these animals were not being fed at all ; they were simply grassed, and were dependent wholly upon the grass, and had been for a period of five or six months prior to the enzootic outbreak that I have referred to. I might also state that observations in Cincinnati by Dr. J. C. Meyer, Sr., indicated that an enzootic outbreak that occurred there one year, covering a limestone district, the year being an exceedingly dry one—might it not be that the dryness of the ground prevented the forage from taking up the usual amount of salts that grass and other forage takes up usually? and, therefore, the disease occur, the animals having fed on that for a long period of time, from not receiving the usual amount of salts necessary to maintain the equilibrium of the body and build up the osseous structures as well as the other tissues of the body?

*Prof. Richardson.*—I am speaking very largely, of course, upon the point that it is a decalcifying process almost entirely. I can well conceive that under certain circumstances—for instance, in seasons of early dryness, the grain ripens rapidly, before it has perfectly matured, and not only would the grain be deficient in phosphates, so essential to the building of bony structure, but it would also certainly be to a greater or less extent deficient in both flourine compounds and in salacious compounds, and in that way the disease may be pre-induced, from a want both of chloride of calcium and from silicates, as well as the tricalcic phosphate.

*The Chair.*—I would like to ask Dr. Bell, of Watertown, whether they are troubled with osteoporosis up his way.

*Dr. Bell*—Not very often. I have seen but two cases in nine years.

*The Chair.*—We also have Dr. Bell, of Erie, with us. We should like to hear from his territory on this subject.

*Dr. Bell*, of Erie.—We have nothing of any account, Mr. President.

*Dr. Sutterby.*—How did the cases terminate that you had, fatally?

*Dr. Bell*, of Watertown—Yes, sir.

*Dr. Sutterby.*—I move that this Society tender Dr. Morris a vote of thanks for the able paper on osteoporosis which he has presented here to-day.

*Dr. Bell*, of Watertown.—I second the motion. (Carried.)

